# Prevalence of and risk factors for bacteremic UTIs in hospitalized adults without definitive signs or symptoms of UTI

**DOI:** 10.1017/ash.2023.251

**Published:** 2023-09-29

**Authors:** Sonali Advani, David Ratz, Jennifer Horowitz, Lindsay Petty, Kenneth Schmader, Tawny Czilok, Anurag Malani, Tejal Gandhi, Scott Flanders, Valerie Vaughn

## Abstract

**Background:** IDSA guidelines recommend withholding treatment in patients with asymptomatic bacteriuria in the absence of systemic signs of infection. However, some patients with bacteriuria may not be able to express symptoms either due to presence of indwelling catheter, underlying complicated urologic anatomy, dementia, or altered mental status (AMS). Clinicians frequently treat bacteriuria in this population with antimicrobial therapy due to concern for sepsis. To determine treatment need, we aimed to review prevalence and risk factors for bacteremic urinary tract infection (UTI) in a cohort of hospitalized inpatients without definitive signs and symptoms of a UTI. **Methods:** This retrospective cohort study of inpatients with a positive urine culture who presented without definitive signs or symptoms of a UTI was conducted between July 1, 2017, and June 30, 2022, in 68 academic and community hospitals (Michigan Hospital Medicine Safety Consortium). Signs and symptoms were obtained from medical record review 3 days before and after urine-culture collection. Bacteremic UTI was defined as any positive blood culture growing at least 1 organism matching the urine culture. Risk factors for bacteremic UTI were assessed using multivariable logistic regression models with results expressed as odds ratios (ORs) for dichotomous variables and relative risks (RRs) for continuous variables. **Results:** Of 11,793 patients meeting study criteria, 73.6% were female with a median age of 78.2 years. Overall, 41.8% had AMS, 33.8% had dementia, 15.6% had an indwelling urinary catheter, and 54.6% had complicated urologic history (eg, urologic surgery). Of these, 166 patients (1.4%) developed bacteremic UTI. On adjusted analysis, male sex, hypotension, heart rate >90, urinary retention, fatigue, log of serum leukocytosis [1 log increase in serum WBC = 2.718 × serum white blood cell count (WBC)], and pyuria with >25 WBC per high-powered field (WBC/hpf) on urinalysis were associated with bacteremic UTI (Table). Older age, presence of an indwelling catheter, complicated urologic history, functional decline, AMS, dementia, and change in urine were not associated with higher odds for bacteremic UTI (Table). Of patients with AMS and no definitive signs or symptoms of a UTI, only 89 (1.8%) of 4,932 developed a bacteremic UTI. **Conclusions:** Bacteremic UTI is relatively rare in hospitalized inpatients presenting with bacteriuria without symptoms of UTI. Predictors of bacteremic UTI included male sex, hypotension, tachycardia, urinary retention, fatigue, serum leukocytosis, and higher levels of pyuria (>25 WBC/hpf) on urinalysis. Our findings provide stewards a framework to risk stratify inpatients of older age who present with positive urine cultures but without (or are unable to express) signs or symptoms of UTI.

**Figure:**
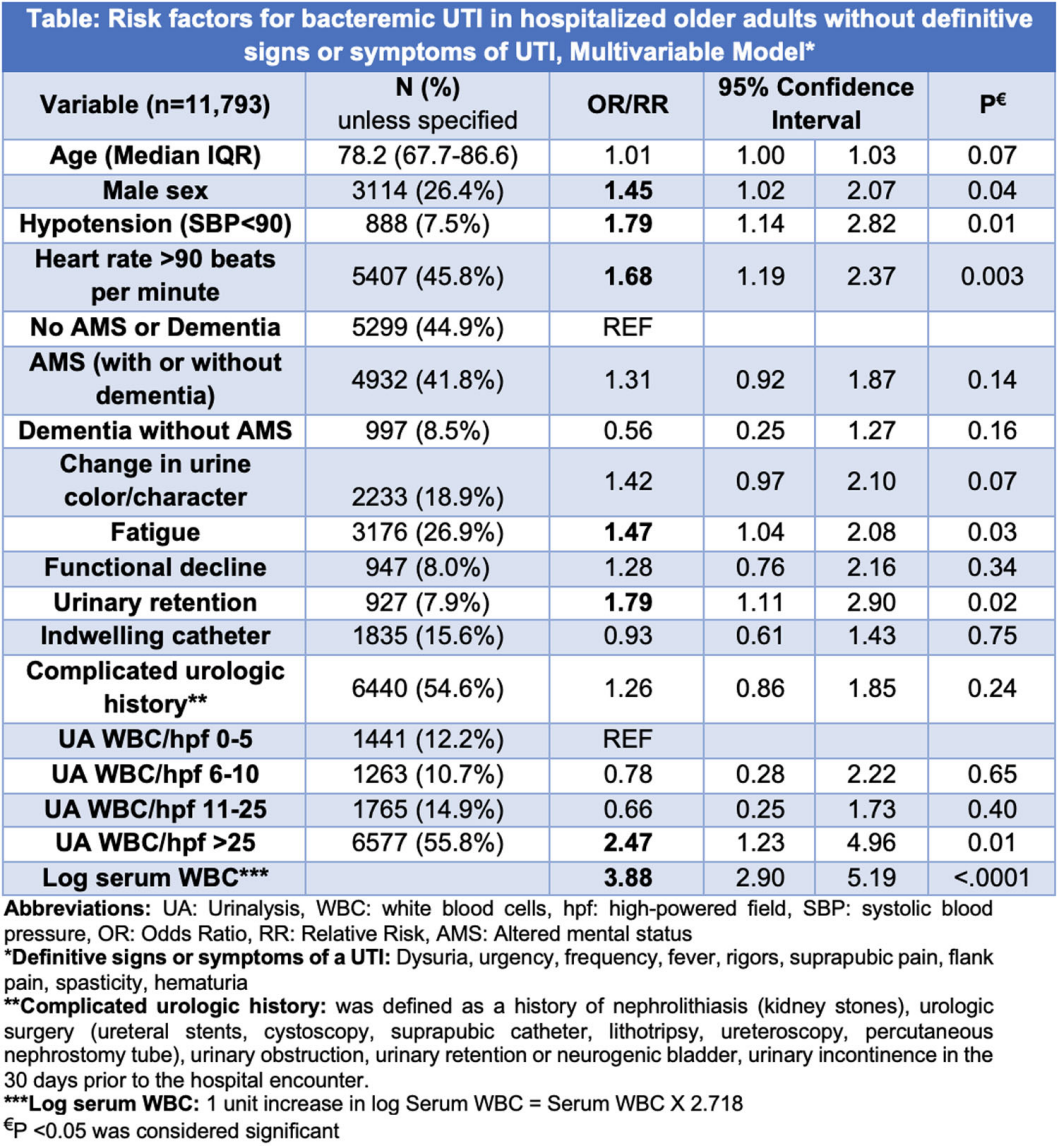

**Disclosures:** None

